# The Role of Guided Bone Regeneration in Enhancing Dental Implant Success: Guided Bone Regeneration in Dental Implant

**DOI:** 10.31661/gmj.v13iSP1.3681

**Published:** 2024-12-08

**Authors:** Emad Taghizadeh, Sahar Negargar, Kosar Nuroozi Larki, Raman Saberi Haghighi, Hossein Shahoon

**Affiliations:** ^1^ Department of Oral and Maxillofacial Surgery, Faculty of Dentistry, Shahed University, Tehran, Iran; ^2^ Department of Dentistry, Asad Abadi Hospital, Tabriz, Iran; ^3^ Department of Restorative Dentistry, School of Dentistry, Ahvaz Jundishapur University of Medical Science, Ahvaz, Iran; ^4^ Department of Dentistry, Tehran University of Medical Sciences, Tehran, Iran

**Keywords:** Guided Bone Regeneration, Dental Implants, Bone Augmentation, Implant Success Rate, Dental Surgery

## Abstract

Guided Bone Regeneration (GBR) has become an essential technique in dental implantology, particularly for cases with compromised bone volume that impact implant success. This narrative review examines the role of GBR in enhancing dental implant outcomes, focusing on its applications, biological principles, materials, and clinical protocols. GBR utilizes barrier membranes and bone graft materials to foster bone regeneration in deficient areas, creating a stable foundation for implant placement by preventing soft tissue invasion and promoting osteogenic activity. Through a literature review of recent studies, we assess the clinical efficacy of GBR in addressing bone insufficiencies resulting from periodontal disease, trauma, or resorption following tooth loss, with a particular emphasis on how GBR augments implant stability and long-term survival. The review explores various GBR materials, including resorbable and non-resorbable membranes, and graft types such as autografts, allografts, xenografts, and synthetic options. Additionally, advancements in bioactive membranes, growth factor-enhanced materials, and 3D-printed scaffolds are discussed for their potential to improve regenerative outcomes and reduce procedural complications. Best practices in clinical protocols, including preoperative planning, precise membrane placement, and post-operative care, are analyzed to highlight factors that enhance GBR success. Comparative analyses indicate that GBR significantly improves implant survival and reduces marginal bone loss, demonstrating its efficacy in complex cases. Despite its high success rate, GBR has limitations, such as the potential for complications like peri-implantitis and membrane exposure. The paper concludes with suggestions for future research on optimizing GBR materials, enhancing biological responses, and improving long-term outcomes to broaden its application in dental implantology. This review serves as a resource for clinicians and researchers seeking to maximize implant success through advanced GBR techniques.

## Introduction

Guided Bone Regeneration (GBR) has emerged as a pivotal technique in modern dental implantology, addressing one of the most critical factors affecting implant success: the quality and quantity of supporting bone [1]. Dental implants have become the standard of care for tooth replacement, providing long-term functional and aesthetic benefits [2]. However, insufficient bone volume due to periodontal disease, trauma, or natural resorption following tooth loss poses a significant challenge for implant stability and osseointegration [3]. GBR is specifically designed to promote bone growth in deficient areas by using barrier membranes and grafting materials that support the formation of new bone while preventing soft tissue invasion [4]. This biological approach not only enables clinicians to achieve successful implant outcomes in complex cases but also expands treatment options for patients previously deemed unsuitable for implants due to bone insufficiencies [3].

The primary aim of this review is to synthesize current research on the role of GBR in enhancing dental implant success, focusing on its applications, efficacy, and advancements in the materials and techniques used. By providing a comprehensive overview, this review intends to inform both clinicians and researchers on the practical benefits of GBR and the mechanisms by which it contributes to improved implant stability, patient satisfaction, and long-term outcomes.

## Materials and Methods

This narrative review synthesizes current research on GBR and its role in enhancing dental implant success. Following a structured approach, the review provides an overview of GBR advancements, clinical protocols, and materials.

### Literature Search Strategy

A comprehensive literature search was conducted using PubMed, Scopus, and Web of Science to capture relevant studies. Keywords included "Guided Bone Regeneration," "dental implants," "bone augmentation," "barrier membranes," and "implant success rate." Searches were limited to English-language articles, focusing primarily on studies from the past ten years to incorporate both foundational knowledge and recent advancements. Reference lists of pertinent articles were reviewed to identify additional critical studies.

### Inclusion and Exclusion Criteria

* Inclusion Criteria: Articles were included if they provided clinically relevant data on GBR in dental implantology, focused on adult human subjects, and covered topics like biological principles, protocols, materials, or success rates. Preference was given to randomized controlled trials, meta-analyses, systematic reviews, and larger studies (≥20 subjects).

* Exclusion Criteria: Studies were excluded if they focused solely on in vitro or animal models, involved fewer than 20 participants, used outdated GBR materials, or did not directly address GBR’s role in dental implant success.

### Data Extraction and Synthesis

Data from relevant studies were organized by thematic areas: (1) biological mechanisms of GBR, (2) clinical protocols and techniques, (3) GBR materials and advancements, and (4) comparative success rates. Findings from randomized controlled trials and meta-analyses were emphasized where applicable. Limitations of this narrative review include potential selection bias and the absence of systematic meta-analysis, though some foundational studies outside the ten-year range were included for context.

## Fundamentals of GBR

GBR is a surgical approach that facilitates new bone growth in areas with insufficient bone volume, supporting the structural integrity needed for successful dental implant placement [5]. This technique is rooted in the principle of selective tissue regeneration, where bone cells are encouraged to proliferate and form new bone while the invasion of faster-growing soft tissue cells is strategically blocked [6]. GBR is typically indicated for patients who present with localized bone defects due to tooth loss, trauma, or periodontal disease, and who require bone augmentation to secure stable implant placement [7].

The core of GBR lies in the use of barrier membranes, which are biocompatible materials designed to physically separate the bone defect site from surrounding soft tissues [5]. By creating this barrier, the membranes prevent epithelial and connective tissue cells from migrating into the bone defect, thus preserving the space for osteogenic cells to populate the area and promote bone regeneration [8].

In addition to barrier membranes, GBR may involve the use of various bone graft materials. Through careful selection and application of membranes and graft materials, GBR supports the gradual formation of a stable, vascularized bone structure capable of supporting dental implants [9]. This process not only augments bone volume but also improves the predictability of implant outcomes, making GBR an essential technique in modern dental implantology [10].

## Materials Used in GBR

**Table:** Table1. Types of GBR Materials and Their Characteristics

**Type of Material**	**Examples**	**Advantages**	**Limitations**
Barrier Membranes	Resorbable, non-resorbable	Resorbable reduces need for removal; non-resorbable provides stability	Risk of infection, material exposure
Bone Graft Materials	Autografts, Allografts, Xenografts, Synthetics	Autografts promote natural healing; allografts are widely available	Limited bone availability (autografts), risk of immune response (xenografts)
Growth Factors and Agents	BMP-2, PRP, PRF	Enhances osteogenic potential	High cost, variable efficacy

The effectiveness of GBR in enhancing bone formation and providing structural support for dental implants largely depends on the careful selection of materials [5]. These include barrier membranes, bone grafts, and biological agents, each contributing uniquely to predictable regeneration outcomes [11]. Understanding the characteristics, advantages, and limitations of each type is crucial for optimal clinical success in GBR [12]. Table-1 provides an overview of materials used in GBR and their respective properties, facilitating comparison.

### Barrier Membranes

Barrier membranes are foundational in GBR, acting as a barrier to prevent soft tissue from invading the bone defect, allowing slower-growing osteogenic cells to populate the site [2]. The two primary types of membranes are resorbable and non-resorbable, each suited to specific clinical scenarios.

Resorbable membranes, often made of collagen, degrade naturally over time, eliminating the need for a second surgery. This reduces patient discomfort and minimizes surgical risks. However, due to their limited durability, resorbable membranes provide less long-term stability and are typically used in smaller defects or areas under minimal mechanical stress [13].

Non-resorbable membranes, like those made from expanded polytetrafluoroethylene (e-PTFE), maintain stability over longer periods and are used in larger or more complex defects. Despite their effectiveness in creating space for bone growth, non-resorbable membranes often require follow-up surgery for removal, introducing additional risks and discomfort. Clinicians choose between resorbable and non-resorbable membranes based on defect size, patient factors, and the desired duration of structural support [14].

### Bone Graft Materials

Bone grafts are frequently paired with membranes to improve regenerative potential. Different types of graft materials bring unique properties to GBR.

• Autografts, sourced from the patient's own body, are highly effective due to their osteoconductive, osteoinductive, and osteogenic qualities, containing live cells and growth factors essential for bone formation. However, autografts are limited by donor site availability and the need for an additional procedure, which can increase patient morbidity [15].

• Allografts, derived from human donors, provide an osteoconductive scaffold and eliminate the need for a secondary surgical site. While they lack live bone cells, modern processing techniques mitigate immune response risks, making them an effective option for supporting new bone growth [2].

• Xenografts, usually sourced from animals (e.g., bovine), are highly biocompatible and provide an osteoconductive matrix for bone growth. Though effective, xenografts are less osteoinductive than autografts and allografts and may integrate more slowly with the host bone [16].

• Synthetic grafts, such as hydroxyapatite and beta-tricalcium phosphate, offer a consistent and controlled structure, acting as a stable osteoconductive scaffold. These materials are safe and free from biological risks, although they lack the osteoinductive and osteogenic properties of natural grafts. Synthetic grafts are useful where bone regeneration can be expected naturally [17][18].

### Biological Agents

Biological agents, including growth factors and platelet-rich plasma (PRP), are increasingly utilized in GBR to enhance bone regeneration by stimulating cellular activity.

• Growth factors, such as bone morphogenetic proteins (BMPs), play a pivotal role in signaling pathways that promote bone formation [19].

• Platelet-rich plasma (PRP) and platelet-rich fibrin (PRF), derived from the patient’s blood, are autologous sources of concentrated growth factors that promote tissue repair [20].

## Advancements in GBR Materials and Technologies

GBR is advancing with new materials and techniques that address the limitations of traditional approaches, resulting in improved outcomes and more predictable bone regeneration at implant sites [21]. Key innovations include bioactive membranes, growth factor-enhanced materials, and 3D-printed scaffolds, all designed to accelerate and enhance bone healing for both clinicians and patients [22].

### Bioactive Membranes

In contrast of traditional barrier membranes that simply block soft tissue ingrowth, bioactive membranes actively support bone regeneration. Embedded with osteoconductive or osteoinductive agents (e.g., calcium phosphates, bioactive glass), these membranes promote bone cell adhesion and proliferation directly at the defect site, leading to faster and more robust bone formation [23]. Additionally, most bioactive membranes are resorbable, eliminating the need for secondary removal surgery, which reduces patient discomfort and infection risks [24].

### Growth Factor-enhanced Materials

GBR materials incorporating growth factors like BMPs and platelet-derived growth factor (PDGF) stimulate cellular activity to accelerate bone formation [19][25]. These growth factors offer controlled, localized release, supporting osteogenic cell migration and differentiation. For instance, BMP-2 significantly improves bone quality and volume when combined with bone grafts or bioactive membranes [26][27].

### 3D-printed Scaffolds

3D printing provides custom-fit scaffolds tailored to specific bone defects. Composed of biocompatible materials such as polycaprolactone (PCL) or hydroxyapatite, these scaffolds optimize fit and stability while reducing movement [22]. 3D Functionalized with bioactive agents, they combine structural support with biological stimulation, proving especially effective in complex cases needing intricate shaping [28][22].

### Clinical Applications and Indications

GBR is primarily indicated in dental implantology for patients with insufficient bone volume, which could arise from various causes, including periodontal disease, trauma, congenital defects, or natural bone resorption following tooth loss [29]. In such cases, GBR is employed to create a stable, augmented bone structure capable of supporting an implant, improving both functional and aesthetic outcomes.

### Indications for GBR

1. Bone loss due to periodontal disease: Periodontal disease is a major contributor to bone resorption in the jaw, often resulting in localized defects [30]. GBR is essential in these cases to regenerate lost bone, restoring adequate volume for implant placement. GBR provides a framework that facilitates bone cell proliferation, reversing periodontal bone loss and enhancing implant stability [31].

2. Bone deficiency after trauma or injury: Facial trauma from accidents or surgical procedures can lead to significant bone loss or deformities, complicating implant placement [32]. GBR techniques are valuable for restoring the damaged bone structure in these cases, enabling optimal implant positioning. In addition to stabilizing the implant, GBR in trauma-related cases often improves facial symmetry and functionality [22].

3. Bone resorption following tooth loss: Natural bone resorption frequently occurs after tooth extraction, often reducing bone volume in the edentulous ridge [33]. GBR is commonly indicated to restore ridge height and width, providing a stable base for implant integration. By regenerating bone in areas of resorption, GBR increases the predictability of implant outcomes and enhances the likelihood of successful osseointegration [34].

### Techniques for Complex Cases Requiring GBR

1. Vertical and horizontal ridge augmentation:

In cases of significant bone loss, GBR is used to augment both the vertical and horizontal dimensions of the ridge [35]. Vertical ridge augmentation restores height, while horizontal augmentation increases width, both essential for implants that require greater support. GBR in these cases helps achieve the ideal implant orientation, enhancing both function and aesthetics [36][37]

2. Sinus lift procedures: When implant placement is limited by the sinus cavity in the posterior maxilla, GBR techniques are often combined with sinus lift procedures to create adequate bone height [38]. By augmenting the bone within the sinus space, GBR facilitates implant stability even in anatomically challenging areas, expanding the scope of possible implant placements [21].

3. Immediate implant placement with simultaneous GBR: For immediate implant placement following extraction, GBR can be performed simultaneously to address existing bone deficits or prevent future resorption. In cases with compromised or thin surrounding bone, GBR offers additional support, creating a stable environment for osseointegration and minimizing the risk of future bone loss around the implant [23].

4. Aesthetic zone implants: In the anterior maxilla, GBR is often necessary to achieve optimal aesthetics by restoring bone to support surrounding gum tissue [39]. Bone augmentation in this area not only improves implant stability but also enhances visual outcomes, particularly in patients with high smile lines where any deficiency is easily visible [2].

## Clinical Protocols and Best Practices in GBR

**Figure-1:**
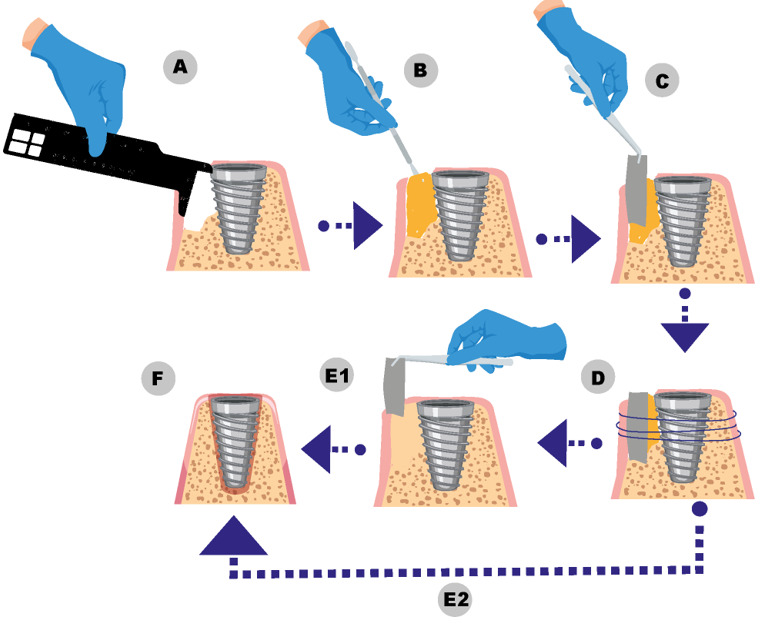
Figure 1. Schematic Diagram of Guided Bone Regeneration Technique A. Measurement of Defect: Using a ruler or measuring tool, the bone defect area around the implant is measured to assess the space needed for guided bone regeneration. B. Placement of Bone Graft Material: A bone graft material is applied to the deficient area around the implant, filling the bone defect. C. Insertion of Guided Tissue Membrane: A guided tissue membrane is inserted to cover the bone graft, helping to stabilize the graft material and provide a barrier to soft tissue invasion. D. Membrane Placement over Graft: A barrier membrane is placed over the bone graft material to protect it and prevent soft tissue from invading the graft area. Sutures or other fixation methods are used to secure the membrane in place. E1. Removal of Non-Absorbable Barrier Membrane: The non-absorbable barrier membrane is removed after the bone regeneration period, allowing inspection of the newly formed bone. E2. Absorbable Barrier Membrane: An absorbable barrier membrane is used, which gradually dissolves over time, eliminating the need for removal and providing prolonged protection for bone regeneration. F. Final Bone Formation and Implant Stability: After the healing period, the bone is fully regenerated around the implant, providing stability and support for the implant within the newly formed bone.

GBR requires a structured approach encompassing preoperative planning, precise surgical execution, and diligent post-operative care to optimize bone regeneration and implant stability [40]. Adherence to best practices across these phases enhances predictable outcomes and minimizes complications.

### Preoperative Assessment

Effective GBR begins with a comprehensive preoperative evaluation to assess bone deficiency, anatomical challenges, and patient-specific risk factors [10]. Imaging, primarily through cone-beam computed tomography (CBCT), provides detailed insights into bone volume, defect morphology, and proximity to anatomical structures like the maxillary sinus and mandibular nerve, enabling precise planning of the augmentation volume and implant positioning [15]. Beyond imaging, a thorough patient evaluation is necessary to identify factors affecting bone healing, including smoking, diabetes, periodontal disease history, and medications (e.g., bisphosphonates) that impact bone metabolism. Recognizing these risk factors allows clinicians to adapt GBR protocols, potentially incorporating additional regenerative materials or adjusting post-operative care [13].

### Surgical Techniques

The surgical phase of GBR involves critical steps designed to establish a stable bone foundation for implants. Figure-1 illustrates the schematic of the step-by-step surgical techniques.

1. Flap Design and Defect Exposure: A full-thickness flap is created to expose the bone defect while preserving the periosteum, crucial for vascular supply [41]. The flap design is essential, with an adequately sized flap allowing access and enabling tension-free closure. Precise incisions and gentle handling of soft tissue minimize trauma, supporting better healing [42].

2. Membrane placement and stabilization: After debridement to remove granulation tissue or infection, bone graft material is placed in the defect as a scaffold for new bone growth [43]. A barrier membrane, either resorbable or non-resorbable, is then positioned over the graft. Membrane stabilization is essential to prevent movement, which can disrupt regeneration. Resorbable membranes are typically fixed with sutures or pins, while non-resorbable membranes may require screws. Keeping the membrane immobile preserves space for bone growth and prevents soft tissue invasion [7].

3. Achieving tension-free closure: Tension-free flap closure is critical to reduce the risk of membrane exposure, a common GBR complication. Mobilizing the flap sufficiently to cover the membrane without tension, using techniques like periosteal scoring or undermining, allows primary closure over the graft. Proper closure shields the graft and membrane from the oral environment, reduces inflammation, and supports smooth healing [44].

### Post-operative Care

Post-operative care is vital for managing inflammation, infection risk, and early bone healing.

1. Medications: To control infection and inflammation, patients typically receive antibiotics (e.g., amoxicillin or clindamycin) and anti-inflammatory medication. NSAIDs are commonly used to manage post-operative discomfort and swelling [13].

2. Oral hygiene and wound care: Patients are advised to maintain oral hygiene without disturbing the surgical site. Antibacterial mouth rinses, such as chlorhexidine, are recommended twice daily to control microbial load, and brushing over the surgical site is avoided for at least two weeks to prevent trauma to healing tissues [45].

3. Follow-up and monitoring: Regular follow-up appointments are critical for tracking healing, detecting early complications, and ensuring graft and membrane stability [46]. Typically scheduled within the first week, then at 2-4 weeks, and at 3 months, follow-ups monitor for membrane exposure, infection signs, or excessive swelling. If non-resorbable membranes are used, they are removed after 4-6 weeks. Long-term follow-ups using radiographic imaging continue to assess bone formation until the site is ready for implant placement [47].

## GBR Success Rates in Implantology

**Table:** Table 2. Summary of Clinical or Meta-analysis Studies on GBR and Dental Implant Survival and Success Rates

**Study**	**Sample Size**	**Type of Membrane**	**Bone Graft Material**	**Success Rate (%)**	**Survival Rate (%)**	**Follow-up (Year)**
Urban et al.[15]	821 I	e-PTFE + CM	Autograft	94.7	100	6
Jung et al.[49]	265 I	e-PTFE or CM	Xenograft	91.9 (CM), 92.6 (e-PTFE)	93.2	12-14
Bazarfshan et al.[50]	73 P	CM	Xenograft	90	97.95	2-7
Cairo et al.[51]	96 P, 195 I	e-PTFE or CM	Autogenous	100	100	5
Roca-Millan et al.[52]	13 studies	Titanium foils	Autograft, Allograft, Xenograft	91.3%	96.5%	1-9
Işık et al.[16]	50 I	CM	Xenograft	100	100	2

**I:** Implants; **P:** Patients; **e-PTFE:** Expanded polytetrafluoroethylene; **CM:** collagen membrane

GBR has been extensively studied for its effectiveness in improving dental implant success, particularly in cases with inadequate bone volume where direct implant placement may be unfeasible [48][49]. Table-2 provides an overview of the membrane types, bone graft materials, and implant success and survival rates.

### Success Rates and Implant Stability

Numerous studies indicate that GBR significantly increases implant success rates, particularly in cases with pre-existing bone deficiencies.[50] A systematic review by Roca-Millan et al., [52], which pooled data from multiple clinical trials and case series, found that the use of titanium membranes in GBR procedures results in comparable bone gain to other commonly used membranes, such as d-PTFE and titanium meshes, with a vertical gain of 7.3 mm and horizontal gains reaching up to 9 mm. Also, they reported the overall implant survival rate and succus rate with GBR was approximately 96.3% and 91.3%, respectively.

Implant stability is another critical factor improved by GBR, as insufficient bone can lead to micromovements that impede initial stability and disrupt osseointegration. Studies indicate that GBR helps to create a denser and more consistent bone structure around the implant, which is vital for both primary and secondary stability [53]. Several studies reported that implants placed with GBR in areas with significant bone loss demonstrated notably higher primary stability and were able to support functional loads sooner than implants placed without GBR. This initial stability reduces the risk of early implant failure and contributes to the overall success of the procedure [54][55][56].

### Osseointegration and Bone Quality

GBR not only supports implant stability but also enhances the quality of osseointegration, the biological process by which the implant surface fuses with the surrounding bone. Successful osseointegration is essential for long-term implant health, as it ensures that the implant remains firmly anchored in the bone. [57, 58 E.,] A systematic review by Esteves et al. [57] analyzed studies on GBR techniques and found that collagen-based membranes enhanced osseointegration by promoting effective bone regeneration around the implant site. The studies showed that collagen membranes facilitate close bone-to-implant contact (BIC), strengthening osseointegration by fostering a stable and supportive bone environment. This enhancement of osseointegration through GBR with collagen membranes indicates their suitability for creating a favorable structure that promotes long-term implant stability [49][57].

Additional studies demonstrate that the bone quality around GBR-treated implants is comparable to that of native bone, further supporting effective osseointegration. By using osteoconductive materials in GBR, such as bone grafts and bioactive membranes, the regenerated bone exhibits similar mechanical properties to surrounding bone. This enhances the implant's resistance to biomechanical forces, reducing the risk of implant loosening or failure over time [59].

## Long-term Survival Rates and Complications

Long-term survival is a primary metric for evaluating the success of dental implants, as implants are expected to provide a permanent solution [55]. A study by Wang et al., [60] reported on the stability of peri-implantitis surgical reconstructive therapy over a 2.5-year period, indicating sustained improvements in probing pocket depth and radiographic marginal bone levels. Also, another study reported a 100% implant survival rate over a follow-up period ranging from 1 to 15 years (mean of approximately 6 years), with satisfactory stability and minimal bone loss observed in most patients [61].

### Bone Stability and Marginal Bone Loss

Maintaining bone stability around implants is crucial for long-term success. GBR not only increases initial bone volume but also appears to support bone preservation over time, reducing marginal bone loss a common indicator of implant health [62]. Studies with follow-up periods indicate that GBR can help maintain alveolar ridge height and prevent significant bone resorption around the implant [60][51]. For instance, A 10-year retrospective cohort study demonstrated that that long-term marginal bone resorption rates in GBR-treated implants were lower compared to non-augmented implants, supporting the role of GBR in mitigating long-term bone resorption [3]. However, the extent of this stability may vary based on factors such as the type of graft material used, membrane properties, and patient-specific factors such as bone density and general health [62].

Despite these positive findings, some degree of bone resorption may still occur over time in GBR sites, potentially due to natural remodeling processes or mechanical stress from the implant. Bone stability is also influenced by the quality and integration of the grafted bone, underscoring the importance of selecting appropriate materials and techniques for each patient to optimize long-term outcomes [61].

### Complications

While GBR offers significant benefits, it is not without complications, particularly in the long term. Peri-implantitis a bacterial infection and inflammation affecting the tissues around the implant is a notable concern in GBR-augmented implants, as it can lead to progressive bone loss and implant failure [63]. Some studies indicate that the risk of peri-implantitis may be slightly higher in augmented sites due to the presence of graft materials, which may create microenvironments more prone to bacterial colonization [60].

Another potential complication is membrane exposure, which can occur if the membrane becomes exposed to the oral environment due to insufficient soft tissue coverage or flap tension [57]. Membrane exposure, particularly in the early stages of healing, poses a risk of infection and may compromise the graft’s success by allowing bacterial invasion [64]. Non-resorbable membranes are especially prone to this complication since they remain in place until removed [65]. Resorbable membranes generally reduce the risk of long-term exposure but may lack the durability needed for extensive defects [10]. Strategies such as tension-free closure and patient-specific flap management help mitigate this risk, but the development of more advanced membranes with enhanced resistance to exposure and bacterial infiltration would be beneficial [66].

## Limitations and Challenges

While GBR is a powerful technique for enhancing implant success, several challenges limit its effectiveness and generalizability in clinical practice. Recognizing these limitations is key to refining GBR approaches and guiding future research in dental implantology [67][68].

### Small Sample Sizes and Study Limitations

Many studies evaluating GBR are conducted with small sample sizes, limiting the generalizability of their findings and creating statistical variability that can complicate reliable conclusions about the effectiveness of specific materials or techniques. This lack of large-scale trials restricts the strength of the available evidence and highlights the need for more robust studies to support clinical decision-making [52]

### Variability in Techniques and Materials

GBR research is complicated by a high degree of variability in techniques and materials across studies. Differences in membrane types (resorbable vs. non-resorbable), bone graft sources (autografts, allografts, xenografts, synthetics), and surgical techniques all impact outcomes, often resulting in inconsistent findings [52][69]. Additionally, variations in membrane placement, fixation methods, and post-operative care protocols add further heterogeneity. Standardization of GBR methods and materials in future studies would improve the comparability of results and enable clearer identification of best practices [69][40].

### Cost and Accessibility

The specialized materials required for GBR, including bioactive membranes, growth factors, and customized scaffolds, contribute to high procedural costs, limiting accessibility in resource-constrained settings [70]. Research focused on developing cost-effective and universally accessible GBR materials could help make this technique a viable option for a broader range of patients, reducing financial barriers to care [71].

### Risk of Complications

Despite its high success rate, GBR is not without risks. Complications such as membrane exposure, infection, and inadequate bone regeneration can occur, potentially prolonging healing, requiring additional interventions, or even leading to implant failure [72]. Advances in materials and surgical techniques have reduced some of these risks, yet the potential for complications remains, particularly in cases with complex anatomical challenges or systemic health issues. Improved patient screening, meticulous preoperative planning, and refined surgical protocols are essential for reducing these risks and improving the predictability of GBR outcomes [15].

## Innovative Directions

Recent advancements in GBR have expanded its applications and improved outcomes, yet research is needed to further enhance materials, biological responses, and long-term success. Focusing on these areas will optimize GBR's potential to support successful dental implants [5].

### Optimizing GBR Materials

Future GBR materials must be biocompatible, and capable of enhancing bone growth. Next-generation membranes that combine mechanical stability with bioactivity such as those releasing osteoinductive factors could improve regeneration efficiency. [71][73][22]. Additionally, hybrid materials blending the durability of non-resorbable membranes with resorbable convenience would reduce the need for secondary surgeries. Affordable options with consistent performance are also vital for broader accessibility, especially in resource-limited settings [9].

### Enhancing Biological Response

The controlled release of growth factors like BMPs, PDGF, and VEGF shows promise in accelerating bone regeneration [27][25]. Future studies should refine using stem or genetically modified cells could create personalized regenerative therapies that align GBR more closely with individual biological profiles, boosting bone growth and reducing healing times [25].

### Digital Integration and 3D Printing

Digital technologies, such as 3D imaging and printing, allow for custom GBR scaffolds and membranes, enhancing fit, stability, and regenerative success [22]. Future 3D-printed scaffolds incorporating bioactive elements could offer integrated solutions that support rapid bone formation [74].

Overall, innovations in GBR materials, biological enhancement, long-term research, personalization, and digital technologies will make GBR more effective, predictable, and accessible, supporting better outcomes in dental implantology.

## Conclusion

GBR has become an indispensable technique in dental implantology, particularly for cases involving compromised bone volume. This review underscores GBR’s effectiveness in enhancing implant success by creating stable, regenerative environments that promote new bone growth. Through the strategic use of barrier membranes and graft materials, GBR facilitates implant stability, enables successful osseointegration, and improves aesthetic outcomes, even in patients with severe bone deficiencies.

The success of this method relies on advancements in materials and techniques, such as resorbable and bioactive membranes, as well as novel grafting materials like autografts, xenografts, and synthetic options. Recent innovations, including 3D-printed scaffolds and growth factor-enhanced biomaterials, show promise for improving GBR’s predictability and reducing patient recovery times. Effective clinical protocols spanning from preoperative planning to post-operative care are essential for minimizing complications and maximizing the benefits of GBR in various implant scenarios, including sinus lifts, ridge augmentation, and aesthetic zone implants.

Despite its success, GBR is not without challenges, including risks of membrane exposure, infection, and peri-implantitis, especially over the long term. Additionally, limitations such as cost, variability in clinical outcomes, and limited long-term studies highlight the need for continued research. Future directions in GBR are likely to focus on optimizing materials for bioactivity, integrating digital technologies for precise implant planning, and personalizing approaches based on patient-specific characteristics. As technology advances, GBR will continue to evolve, offering more predictable, efficient, and accessible solutions that align with the growing demands of modern dental implantology.

## Conflict of Interest

None.
